# Nonlinear association between albumin-globulin ratio and bone mineral density: A cross-sectional study revealing threshold effects and sexual dimorphism in US adults

**DOI:** 10.1097/MD.0000000000050541

**Published:** 2026-09-11

**Authors:** Songlang Liu, Xia Li, Yunhao Wang, Jiewen Wei

**Affiliations:** a Department of Sports Medicine, Affiliated Meizhou Hospital of Shantou University Medical College, Meizhou, China; b Institute of Basic Medical Sciences, Affiliated Meizhou Hospital of Shantou University Medical College, Meizhou, China; c Shantou University Medical College, Shantou, China; d Department of Hand Surgery, Huizhou Sixth People’s Hospital, Huizhou, China.

**Keywords:** albumin-globulin ratio, bone mineral density, NHANES, sexual dimorphism, threshold effect

## Abstract

The albumin-globulin ratio (AGR), which integrates nutritional and inflammatory biomarkers, may reflect skeletal health status; however, its relationship with bone mineral density (BMD) remains inadequately characterized. This cross-sectional analysis utilized data from 10,101 US adults in the National Health and Nutrition Examination Survey 2005 to 2018. Weighted multivariable linear regression was used to evaluate the AGR-BMD associations at the femoral neck and lumbar spine (L1–L4). Generalized additive models identified nonlinearity, with threshold effects quantified by piecewise regression. Subgroup analyses were used to test interactions by sex, race, and comorbidities. After full adjustment, each unit increase in AGR correlated with reduced femoral neck BMD (β = −0.0354 g/cm^2^, 95% confidence interval: −0.0464 to −0.0243) and lumbar BMD (β = −0.0245 g/cm^2^, 95% confidence interval: −0.0369 to −0.0120). Nonlinear analyses revealed inflection points at AGR = 1.7407 (femoral) and 1.1714 (lumbar), with differential associations below or above thresholds. Women exhibited a 3.1-fold stronger inverse association than men (*P*_interaction_ < .0001). Elevated AGR was independently associated with reduced BMD in adults in the United States, demonstrating nonlinear threshold patterns (1.74 for femoral and 1.17 for lumbar BMD) and pronounced sexual dimorphism. These findings suggest that AGR is a composite biomarker that reflects inflammatory-nutritional pathways that are relevant to skeletal homeostasis.

## 1. Introduction

Osteoporosis (OP), a systemic skeletal disorder characterized by diminished bone mineral density (BMD) and impaired bone microarchitecture,^[1]^ significantly increases the risk of fracture. This results in functional impairment, loss of independence, substantial healthcare expenditures, and increased morbidity and mortality.^[2,3]^ Global demographic aging is projected to escalate the burden of OP, with annual direct costs in the United States estimated to reach $25.3 billion by 2025.^[4]^ Consequently, OP represents a critical public health challenge with profound implications for healthcare systems and socioeconomic structures.^[5]^

Emerging evidence underscores the multifactorial pathogenesis of OP, involving inflammatory, immune, and nutritional mediators in its etiology, progression, and outcomes.^[1,6]^ Recent studies have linked systemic inflammatory, nutritional, and metabolic indicators to BMD or OP-related outcomes.^[7–9]^ The albumin-globulin ratio (AGR), calculated as serum albumin divided by serum globulin, serves as a composite metric that reflects integrated systemic information. Serum albumin correlates with nutritional status and inflammation,^[10]^ whereas globulin encompasses acute-phase proteins whose levels increase during inflammatory responses.^[2,11]^ Recent studies have also reported associations of systemic immune-inflammatory indicators and AGR with BMD outcomes.^[7–9]^ Thus, AGR provides a consolidated indicator of systemic inflammation and nutritional state.^[12,13]^ A recent National Health and Nutrition Examination Survey (NHANES) study identified a nonlinear association between AGR and thoracic spine BMD in adolescents^[14]^; however, evidence regarding femoral neck and lumbar spine BMD in adults remains limited.

Utilizing data from the NHANES, this cross-sectional study aimed to assess the association between the serum albumin-to-globulin (A/G) ratio and BMD. We leveraged the robust methodology and extensive covariate data of NHANES to elucidate clinically relevant associations, thereby contributing to the scientific basis of OP prevention strategies.

## 2. Methods

### 2.1. Study design and data source

This cross-sectional investigation utilized data from the NHANES, a nationally representative assessment of health and nutritional status in the United States. Conducted by the National Center for Health Statistics, NHANES employs a multistage stratified probability sampling design across biennial cycles. The National Center for Health Statistics Research Ethics Review Board approved all protocols, and informed consent was obtained from all participants. Publicly accessible data and documentation are available through the NHANES repository. The study received an exempt determination from the Institutional Review Board of Affiliated Meizhou Hospital of Shantou University Medical College owing to the exclusive use of fully deidentified patient records from the NHANES dataset. This study adhered to the Strengthening the Reporting of Observational Studies in Epidemiology guidelines for observational research.

### 2.2. Study population

**Figure 1.**
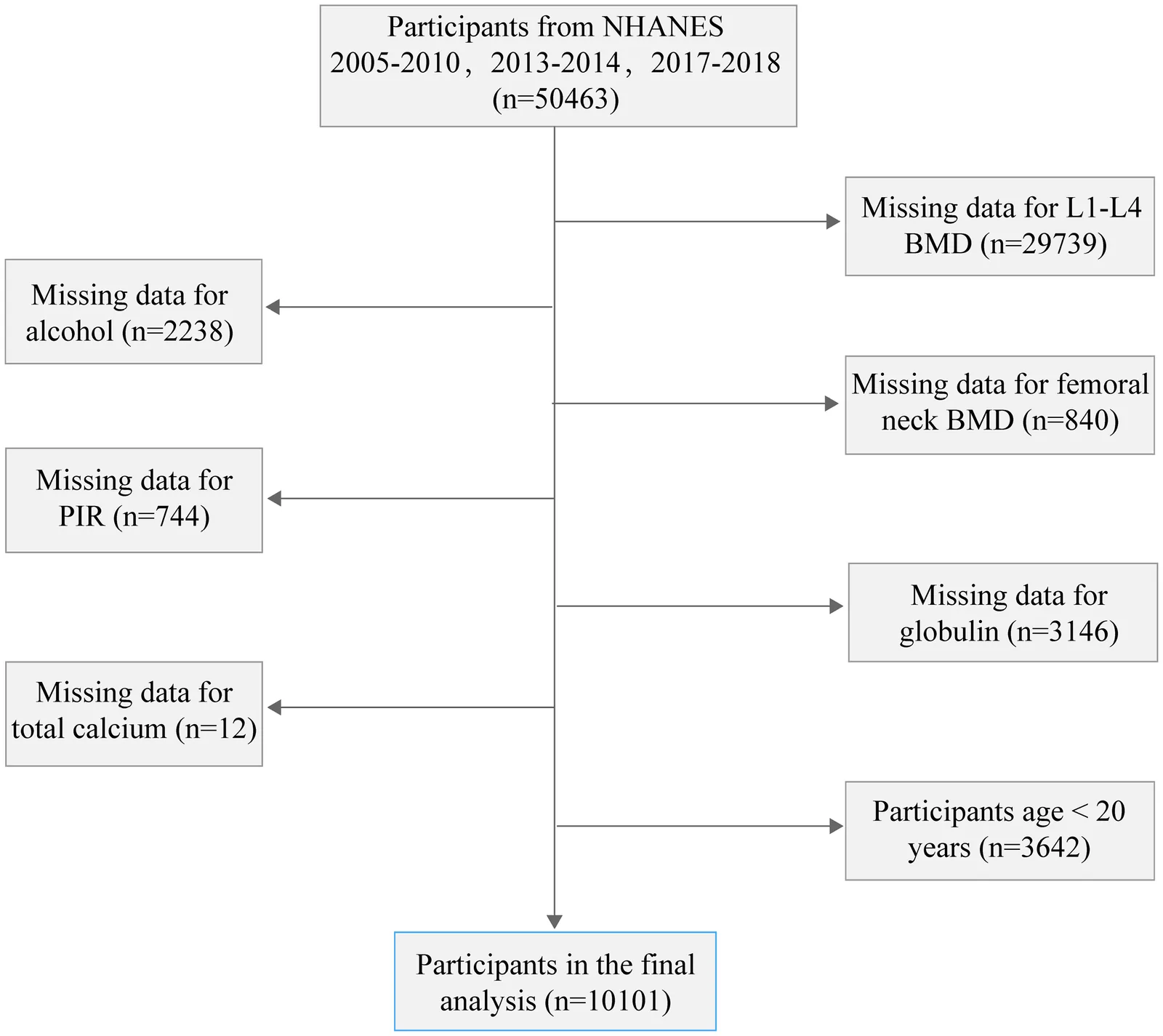
Participant inclusion flowchart. NHANES cycles: 2005 to 2010, 2013 to 2014, and 2017 to 2018. Exclusion criteria were missing lumbar BMD (n = 29,739), missing femoral neck BMD (n = 840), missing globulin (n = 3146), alcohol/PIR/calcium data deficiency (n = 2994), and age <20 years (n = 3642). BMD = bone mineral density, NHANES = National Health and Nutrition Examination Survey, PIR = poverty-income ratio.

We analyzed 5 consecutive NHANES cycles (2005–2006 to 2017–2018, Fig. 1). The exclusion criteria included missing data for lumbar spine (L1–L4) BMD measurements (n = 29,739), femoral neck BMD (n = 840), serum globulin concentrations (n = 3146), alcohol consumption status (n = 2238), poverty-income ratio (PIR, n = 744), total serum calcium (n = 12), and age <20 years (n = 3642). After exclusion, the final analytical cohort comprised 10,101 adults (Fig. 1).

### 2.3. Exposure assessment

AGR served as the primary exposure variable and was calculated as serum albumin (g/dL) divided by serum globulin (g/dL). Serum albumin concentrations were determined via a bromocresol purple binding assay at 600 nm, while total protein concentrations were measured using the Biuret method at 545 nm. Both analyses employed Beckman UniCel DxC800 Synchron (Beckman Coulter, Brea) instrumentation. Globulin concentrations were derived by subtracting albumin from the total protein.^[15]^

### 2.4. Outcome measurement

Dual-energy X-ray absorptiometry was used to quantify the BMD at the femoral neck and lumbar spine (L1–L4 vertebrae). Certified radiology technicians performed all scans following standardized NHANES protocols with rigorous quality control procedures to ensure measurement precision.^[16]^ BMD values were reported in g/cm^2^.

### 2.5. Covariate specification

Adjustment covariates included demographic factors (age, sex, and race/ethnicity), socioeconomic indicators (PIR, educational attainment, and marital status), lifestyle variables (smoking history defined as ≥100 lifetime cigarettes, alcohol consumption categorized as ≥4 drinks/day), clinical conditions (diabetes and hypertension), and serum biomarkers (total calcium, phosphorus, total protein, and creatinine). Comprehensive measurement methodologies for all covariates are documented in the NHANES protocols.

### 2.6. Statistical analysis

All analyses incorporated NHANES sampling weights to account for the complex survey design. Weighted descriptive statistics were used to characterize participants across AGR quartiles. Between-group comparisons were performed using χ^2^ tests for categorical variables and weighted linear regression models for continuous measures.

Multivariable linear regression examined the associations between continuous AGR and site-specific BMD through 3 sequential adjustment models: Model 1 (unadjusted), Model 2 (adjusted for age, sex, and race/ethnicity), and Model 3 (fully adjusted for all specified covariates). Sensitivity analyses treated AGR as categorical (quartiles).

Nonlinear relationships were assessed using weighted generalized additive models with smoothing splines. Threshold effects were quantified via piecewise linear regression with inflection points determined using recursive algorithms. Stratified analyses evaluated potential effect modification by sex, race/ethnicity, diabetes, and hypertension status, testing interaction terms within fully adjusted models.

Statistical computations were performed using R version 4.2.3 (R Foundation for Statistical Computing, Vienna, Austria) and EmpowerStats version 4.2 (X&Y Solutions, Inc., Boston), with two-sided *P*-values <.05 considered statistically significant.

## 3. Results

### 3.1. Baseline characteristics

The analytical cohort comprised 10,101 adults (mean age 48.73 ± 16.15 years; 53.7% male) with complete data. As detailed in Table 1, participants in higher AGR quartiles were predominantly non-Hispanic White males exhibiting higher education levels and socioeconomic status (PIR: 3.51 ± 1.54 vs 3.79 ± 1.65; *P* < .0001). This group demonstrated a lower prevalence of diabetes (4.5% vs 13.2%) and hypertension (21.7% vs 36.8%), along with reduced mean BMD measurements (femoral neck: 0.82 ± 0.14 g/cm^2^ vs 0.84 ± 0.15 g/cm^2^; lumbar spine: 1.02 ± 0.14 g/cm^2^ vs 1.05 ± 0.15 g/cm^2^; both *P* < .0001).

**Table 1:** Weighted characteristics by albumin-globulin ratio quartiles.

AGR	Q1 (0.338–1.312)	Q2 (1.312–1.483)	Q3 (1.483–1.68)	Q4 (1.68–4.5)	*P* value
Age (yr)	48.64 ± 15.23	47.12 ± 15.15	46.63 ± 15.00	45.07 ± 14.59	<.0001
Sex (%)					<.0001
Male	38.60	44.48	53.94	61.79	
Female	61.40	55.52	46.06	38.21	
Race/ethnicity (%)					<.0001
Mexican American	12.12	9.48	7.96	4.32	
Other Hispanic	7.10	5.40	4.16	2.70	
Non-Hispanic White	51.34	66.95	75.81	86.31	
Non-Hispanic Black	22.58	11.77	6.02	2.62	
Other race – including multiracial	6.86	6.40	6.05	4.05	
PIR	3.79 ± 1.65	3.11 ± 1.63	3.26 ± 1.62	3.51 ± 1.54	<.0001
Education level (%)					<.0001
<High school	21.97	16.37	15.05	10.19	
High school	26.74	24.65	23.26	21.37	
>High school	51.29	58.98	61.69	68.44	
Marital status					<.0001
Married/live with partner	61.01	65.67	68.09	70.16	
Never married	15.50	14.40	13.86	15.62	
Separated/divorced/widowed	23.49	19.93	18.05	14.22	
Smoked at least 100 cigarettes in life (%)					.0008
Yes	46.64	46.28	51.50	50.83	
No	53.36	53.72	48.50	49.17	
Alcoholic ≥4 drinks/d					.5487
Yes	17.34	15.18	15.93	16.12	
No	82.66	84.82	84.07	83.88	
Diabetes (%)					<.0001
Yes	13.19	7.16	6.08	4.46	
No	86.81	92.84	93.92	95.54	
Hypertension (%)					<.0001
Yes	36.84	29.25	27.16	21.70	
No	63.16	70.75	72.84	78.30	
Total protein (g/dL)	73.66 ± 4.71	71.70 ± 4.27	70.78 ± 3.90	68.93 ± 3.75	<.0001
Total calcium (mg/dL)	2.34 ± 0.09	2.35 ± 0.09	2.36 ± 0.09	2.38 ± 0.09	<.0001
Serum phosphorus (mg/dL)	1.19 ± 0.18	1.20 ± 0.18	1.21 ± 0.18	1.24 ± 0.18	<.0001
Creatinine (mg/dL)	0.89 ± 0.55	0.87 ± 0.25	0.89 ± 0.23	0.92 ± 0.18	<.0001
Femoral neck BMD (g/cm^2^)	0.84 ± 0.15	0.83 ± 0.15	0.83 ± 0.15	0.82 ± 0.14	.0010
L1–L4 BMD (g/cm^2^)	1.05 ± 0.15	1.04 ± 0.15	1.03 ± 0.14	1.02 ± 0.14	<.0001

Data are presented as mean ± SD or percentage. *P*-values were calculated using weighted linear regression (continuous variables) or χ^2^ tests (categorical variables). BMD values are reported in g/cm^2^.

AGR = albumin-globulin ratio, BMD = bone mineral density, PIR = poverty-income ratio.

The AGR, as an integrated metric, primarily reflects the relative balance between albumin and globulin rather than the presence of absolute clinical hyperalbuminemia or hypoglobulinemia, which were rare in this general population cohort. This ratio effectively captures subclinical inflammatory and nutritional states. While segregation based on strict clinical cutoffs was not feasible due to sample size constraints, our subsequent threshold effect analysis inherently identifies and characterizes subpopulations across the AGR spectrum, serving a similar analytical purpose.

### 3.2. Primary associations

Multivariable linear regression revealed significant inverse associations between continuous AGR and BMD after full adjustment (Model 3): femoral neck BMD: β = −0.0354 g/cm^2^ per unit AGR (95% confidence interval [CI]: −0.0464 to −0.0243; *P* < .0001) and lumbar spine (L1–L4) BMD: β = −0.0245 g/cm^2^ (95% CI: −0.0369 to −0.0120; *P* < .0001). When analyzed categorically (Table 2), participants in the highest AGR quartile showed significantly lower BMD than those in the lowest quartile (femoral neck: β = −0.0334, 95% CI: −0.0422 to −0.0246; lumbar spine: β = −0.0210, 95% CI: −0.0310 to −0.0110; both *P* < .0001).

**Table 2:** Association of continuous and categorical AGR with bone mineral density.

Exposure	Model 1 β (95% CI)	Model 2 β (95% CI)	Model 3 β (95% CI)
Femoral neck BMD (g/cm^2^)			
AGR	−0.0142 (−0.0244 to −0.0040)	−0.0372 (−0.0465 to −0.0280)	−0.0354 (−0.0464 to −0.0243)
AGR categories			
Q1	Reference	Reference	Reference
Q2	−0.0097 (−0.0188 to −0.0006)	−0.0124 (−0.0201 to −0.0046)	−0.0115 (−0.0195 to −0.0036)
Q3	−0.0136 (−0.0223 to −0.0048)	−0.0206 (−0.0282 to −0.0130)	−0.0195 (−0.0276 to −0.0115)
Q4	−0.0170 (−0.0255 to −0.0085)	−0.0340 (−0.0416 to −0.0264)	−0.0334 (−0.0422 to −0.0246)
*P*-value for trend	<.001	<.001	<.001
L1–L4 BMD (g/cm^2^)			
AGR	−0.0189 (−0.0290 to −0.0088)	−0.0364 (−0.0451 to −0.0241)	−0.0245 (−0.0369 to −0.0120)
AGR categories			
Q1	Reference	Reference	Reference
Q2	−0.0029 (−0.0114 to 0.0056)	−0.0041 (−0.0122 to 0.0041)	−0.0025 (−0.0109 to 0.0058)
Q3	−0.0156 (−0.0239 to −0.0072)	−0.0192 (−0.0274 to −0.0109)	−0.0155 (−0.0244 to −0.0067)
Q4	−0.0138 (−0.0222 to −0.0053)	−0.0245 (−0.0332 to −0.0158)	−0.0210 (−0.0310 to −0.0110)
*P*-value for trend	<.001	<.001	<.001

Model 1: unadjusted; Model 2: adjusted for age, sex, and race/ethnicity; Model 3: fully adjusted (covariates detailed in the Methods). β coefficients represent changes in BMD (g/cm^2^).

AGR = albumin-globulin ratio, BMD = bone mineral density, CI = confidence interval.

### 3.3. Effect modification

Sex-stratified analyses demonstrated significant interaction effects (*P* < .0001). As shown in Table 3, inverse AGR-BMD associations were markedly stronger in women: femoral neck: β = −0.0567 (95% CI: −0.0717 to −0.0416) and lumbar spine: β = −0.0565 (95% CI: −0.0717 to −0.0412), compared with men: femoral neck: β = −0.0183 (95% CI: −0.0321 to −0.0045) and lumbar spine: β = −0.0169 (95% CI: −0.0307 to −0.0032).

**Table 3:** Stratified analyses of AGR-BMD associations.

Characteristics	β (95% CI)	*P* for interaction
L1–L4 BMD (g/cm^2^)		
Subgroup analysis stratified by sex		.0001
Men	−0.0169 (−0.0307 to −0.0032)	
Women	−0.0565 (−0.0717 to −0.0412)	
Subgroup analysis stratified by race/ethnicity		.1264
Mexican American	−0.0465 (−0.0962 to 0.0033)	
Other Hispanic	−0.0100 (−0.0752 to 0.0551)	
Non-Hispanic White	−0.0259 (−0.0400 to −0.0119)	
Non-Hispanic Black	−0.0127 (−0.0601 to 0.0347)	
Other race – including multiracial	0.0463 (−0.0103 to 0.1029)	
Subgroup analysis stratified by diabetes		.3380
Yes	−0.0449 (−0.0900 to 0.0002)	
No	−0.0220 (−0.0350 to −0.0091)	
Subgroup analysis stratified by hypertension		.3652
Yes	−0.0154 (−0.0387 to 0.0079)	
No	−0.0281 (−0.0427 to −0.0134)	
Femoral neck BMD (g/cm^2^)		
Subgroup analysis stratified by sex		<.0001
Men	−0.0183 (−0.0321 to −0.0045)	
Women	−0.0567 (−0.0717 to −0.0416)	
Subgroup analysis stratified by race/ethnicity		.1228
Mexican American	−0.0540 (−0.0981 to −0.0100)	
Other Hispanic	−0.0565 (−0.1139 to 0.0010)	
Non-Hispanic White	−0.0371 (−0.0496 to −0.0247)	
Non-Hispanic Black	−0.0395 (−0.0814 to 0.0024)	
Other race – including multiracial	0.0618 (0.0123–0.1114)	
Subgroup analysis stratified by diabetes		.1949
Yes	−0.0566 (0.0006 to 0.0073)	
No	−0.0330 (−0.0443 to −0.0217)	
Subgroup analysis stratified by hypertension		.7863
Yes	−0.0331 (−0.0513 to −0.0149)	
No	−0.0359 (−0.0483 to −0.0235)	

Interaction *P*-values from multiplicative terms in fully adjusted models. Reference group: Q1 AGR.

AGR = albumin-globulin ratio, CI = confidence interval.

No significant interactions were observed for race/ethnicity, diabetes, or hypertension (all *P* > .05).

### 3.4. Nonlinear relationships

**Figure 2.**
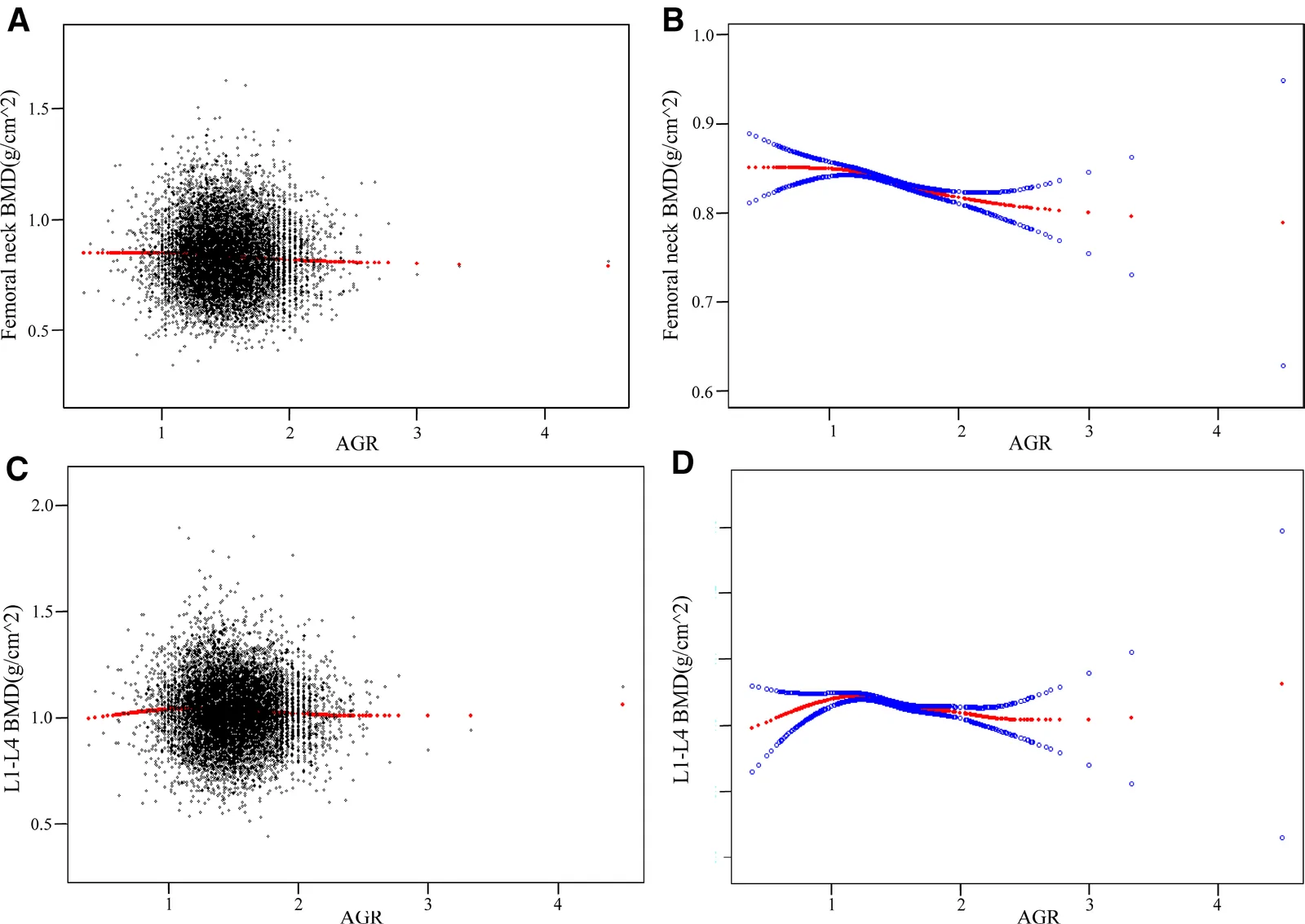
Nonlinear associations between albumin-globulin ratio and site-specific bone mineral density. (A) Scatterplot of AGR and femoral neck BMD; (B) smooth curve fit of AGR and femoral neck BMD; (C) scatterplot of AGR and L1 to L4 BMD; (D) smooth curve fit of AGR and L1 to L4 BMD. Black points represent individual participants. Red lines indicate fitted curves, and blue bands indicate 95% CIs. All adjusted models included the covariates specified in Model 3. AGR = albumin-globulin ratio, BMD = bone mineral density, CI = confidence interval.

**Figure 3.**
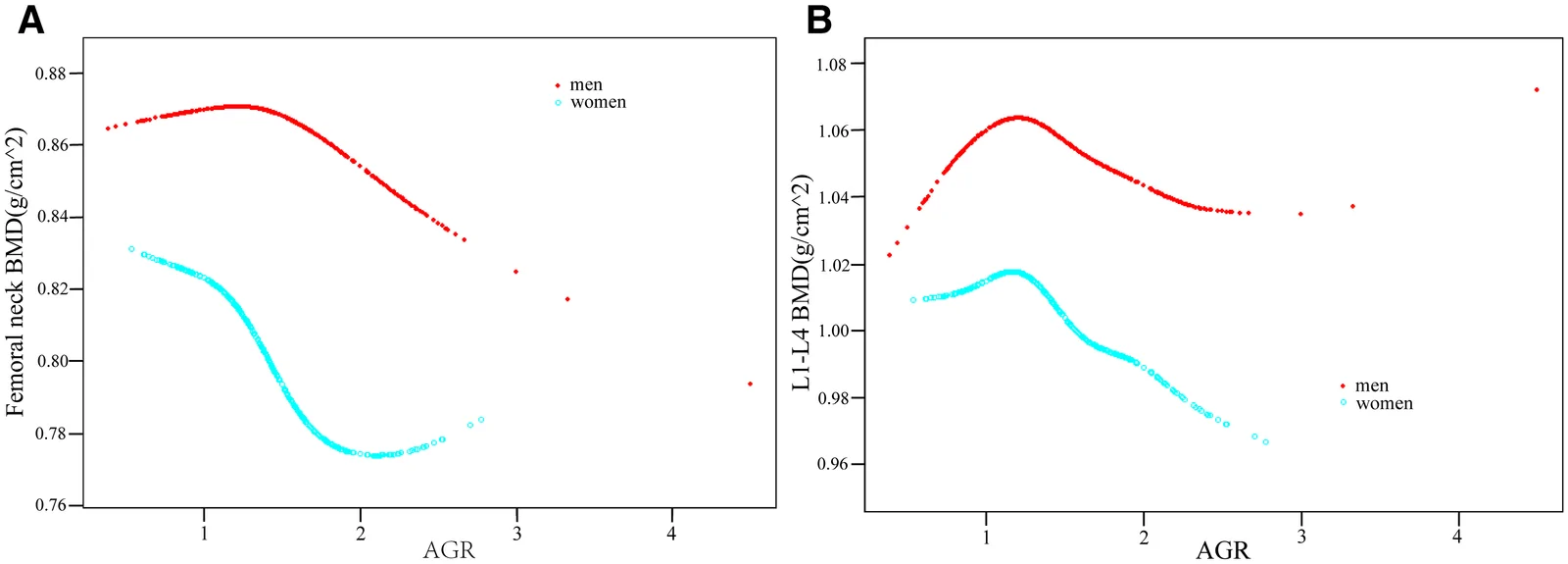
Sex-stratified relationships between albumin-globulin ratio and bone mineral density. (A) Association between AGR and femoral neck BMD stratified by sex; (B) association between AGR and L1 to L4 BMD stratified by sex. AGR = albumin-globulin ratio, BMD = bone mineral density.

Generalized additive models identified nonlinear patterns between the AGR and BMD (Fig. 2). Threshold analyses determined inflection points at AGR = 1.7407 for the femoral neck BMD (log-likelihood ratio, *P* = .004) and AGR = 1.1714 for the lumbar BMD (*P* = .003). Below these thresholds, each unit of AGR increase corresponded to −0.0509 g/cm^2^ femoral BMD (95% CI: −0.0662 to −0.0357) and +0.0825 g/cm^2^ lumbar BMD (95% CI: 0.0098–0.1551), with attenuated associations above these points (Table 4). Sex-stratified curves revealed distinct trajectories (Fig. 3), particularly at AGR < 1.25 (men) and AGR = 1.7 to 2.0 (women).

**Table 4:** Threshold effect analysis of AGR on bone mineral density.

	Model: saturation effect analysis (β [95% CI] [*P* value])
Femoral neck BMD (g/cm^2^)	
AGR turning point (K)	1.7407
<K, effect 1	−0.0509 (−0.0662 to −0.0357) (<.001)
>K, effect 2	−0.0173 (−0.0290 to −0.0055) (.004)
Log-likelihood ratio	0.004
Subgroup analysis stratified by sex	
AGR turning point for men	1.2424
<K, effect 1	0.0797 (0.0056–0.1538) (.035)
>K, effect 2	−0.0265 (−0.0426 to −0.0104) (<.001)
Log-likelihood ratio	0.007
AGR turning point for women	1.72
<K, effect 1	−0.0836 (−0.1049 to −0.0623) (<.001)
>K, effect 2	0.0279 (−0.0103 to 0.0662) (.153)
Log-likelihood ratio	<0.001
L1–L4 BMD (g/cm^2^)	
AGR turning point (K)	1.1714
<K, effect 1	0.0825 (0.0098–0.1551) (.026)
>K, effect 2	−0.0316 (−0.0444 to −0.0187) (<.001)
Log-likelihood ratio	0.003
Subgroup analysis stratified by sex	
AGR turning point for men	1.2424
<K, effect 1	0.1414 (0.0603–0.2226) (<.001)
>K, effect 2	−0.0043 (−0.0219 to 0.0133) (.632)
Log-likelihood ratio	<0.001
AGR turning point for women	1.7083
<K, effect 1	−0.0517 (−0.0762 to −0.0272) (<.001)
>K, effect 2	−0.0046 (−0.0467 to 0.0375) (.830)
Log-likelihood ratio	0.090

β coefficients represent BMD changes (g/cm^2^) per unit AGR below and above the identified inflection points. Log-likelihood ratio tests were considered significant.

AGR = albumin-globulin ratio, BMD = bone mineral density, CI = confidence interval.

## 4. Discussion

### 4.1. Principal findings

This large-scale cross-sectional investigation demonstrated a significant inverse association between the AGR and BMD at clinically relevant skeletal sites among adults.^[1,3,7]^ Following comprehensive adjustment for demographic, socioeconomic, and clinical confounders,^[4–6]^ elevated AGR consistently correlated with reduced femoral neck and lumbar spine BMD. Our analyses further revealed a nonlinear relationship characterized by threshold effects and marked sexual dimorphism, with inverse associations demonstrating a substantially greater magnitude in women than in men. Recent NHANES studies have also linked the systemic immune-inflammation index to BMD in postmenopausal women^[7]^ and AGR to thoracic spine BMD in adolescents.^[14]^

### 4.2. Contextualizing existing evidence

Our observations align with emerging research linking inflammatory and nutritional biomarkers to skeletal homeostasis. Recent population-based studies have similarly linked systemic immune-inflammatory indicators and AGR with BMD.^[7–9]^ Prior investigations have predominantly examined isolated components of the AGR system, notably the osteogenic properties of albumin in bone matrix applications^[4,17,18]^ and the association of sex hormone-binding globulin with BMD.^[19,20]^ The current study extends this literature by evaluating an integrated AGR metric.^[11–14]^ The inverse relationship we observed contrasts with reports indicating that hypoalbuminemia correlates with reduced BMD,^[20–22]^ suggesting that the composite nature of the ratio captures distinct pathophysiological processes.^[21,23–26]^ This apparent discrepancy underscores the complexity of protein metabolism in bone homeostasis, wherein both deficiency and excess may exert detrimental effects.^[10,11]^

### 4.3. Potential mechanistic pathways

The association between AGR and BMD may involve interconnected biological pathways. Hypoglobulinemia, particularly gamma-globulin deficiency, compromises anti-inflammatory capacity,^[23,25,27]^ potentially triggering pro-inflammatory cytokine release that accelerates osteoclastogenesis via nuclear factor kappa B activation.^[24,28,29]^ Concurrent reductions in antioxidant globulin components (e.g., ceruloplasmin) may induce oxidative stress-mediated osteoblast apoptosis.^[12,30]^ Pathological hyperalbuminemia from dehydration or hypercortisolism can impair bone microcirculation^[31]^ and suppress osteoblast activity.^[32–34]^ Globulin deficiency may simultaneously restrict the availability of essential amino acids for collagen synthesis and disrupt insulin-like growth factor 1 bioavailability by reducing binding proteins.^[35,36]^ These pathways provide biologically plausible explanations for the observed association. However, they were not directly evaluated in the present study and should be regarded as hypotheses rather than established mechanisms.^[37–39]^

### 4.4. Interpretation of nonlinearity and threshold effects

The observed nonlinear associations characterized by distinct inflection points (AGR = 1.74 for femoral neck and 1.17 for lumbar spine BMD) suggest a complex, biphasic relationship that may arise from a shift in the dominant pathophysiological pathway influencing bone metabolism at different AGR levels.^[29,30]^ At lower AGR levels (indicating relative hypoalbuminemia and/or hyperglobulinemia), inflammatory pathways might be relatively more important, with elevated globulin fractions amplifying the pro-inflammatory cytokine cascade, promoting osteoclastogenesis and bone resorption. Conversely, at higher AGR levels (indicating relative hyperalbuminemia and/or hypoglobulinemia), the nutritional-endocrine and potential hemodynamic pathways may become more relevant, with extreme albumin dominance potentially disrupting bone microcirculation, osteoblast function, and the bioavailability of anabolic hormones and growth factors bound to globulins (e.g., insulin-like growth factor 1).^[28,29,35,36]^ Such a transition might partly explain the changing slope of the AGR-BMD association. However, this interpretation remains hypothetical because the relevant pathways were not directly evaluated.

### 4.5. Sex-specific modulatory effects

The pronounced sex disparity in effect magnitude warrants mechanistic considerations. Women exhibited approximately threefold larger inverse association estimates than men, which may partly reflect sex-related differences in hormonal regulation and protein metabolism.^[40]^ Differences in estrogen status may influence albumin and globulin metabolism, potentially contributing to the observed association in women.^[22,24]^ Furthermore, estrogen’s direct osteoprotective effects may create a physiological backdrop where any disruptive influence on bone homeostasis, such as that potentially signaled by an elevated AGR, is more acutely manifested. Sexual dimorphism in body composition, immune response, and hepatic protein synthesis may further contribute to these differential associations. The distinct nonlinear patterns identified, with lower inflection points in men (AGR = 1.24) than in women (AGR = 1.71), are consistent with biologically divergent regulatory mechanisms between sexes.^[25,26]^ Nevertheless, these explanations remain hypothetical because hormonal, inflammatory, and protein-metabolism pathways were not directly evaluated in this study.^[41]^

### 4.6. Methodological considerations

The strengths of this study include nationally representative sampling,^[16]^ standardized dual-energy X-ray absorptiometry protocols,^[16]^ and rigorous covariate adjustment adhering to the Strengthening the Reporting of Observational Studies in Epidemiology guidelines. Limitations necessitate cautious interpretation: The cross-sectional design precludes causal inference and cannot exclude reverse causation pathways. Residual confounding from unmeasured factors such as detailed micronutrient intake and physical activity (despite our adjustment for body mass index and general physical activity level) remains possible. The absence of bone turnover markers limits mechanistic insight, while identified inflection points require prospective validation across diverse populations.^[5,6,41]^ Most importantly, the cross-sectional design precludes causal inference and cannot establish the predictive utility of AGR for OP onset or progression. The associations identified are hypothesis-generating; prospective longitudinal studies are essential to determine whether monitoring AGR can serve as a viable tool for predicting future bone loss or fracture risk.

## 5. Conclusion

This study identified an inverse association between serum AGR and BMD in adults in the United States, persisting after multivariable adjustment. Elevated AGR was correlated with reduced femoral neck and lumbar spine BMD, exhibiting nonlinear threshold effects and stronger inverse associations in women than in men. These findings suggest that AGR may be a composite marker associated with skeletal health, although the underlying inflammatory and nutritional pathways remain to be established. Study limitations preclude causal inference, and prospective validation of inflection points is warranted.

## Author contributions

**Conceptualization:** Songlang Liu, Xia Li, Jiewen Wei.

**Data curation:** Songlang Liu, Xia Li, Jiewen Wei.

**Formal analysis:** Songlang Liu, Xia Li, Jiewen Wei.

**Funding acquisition:** Yunhao Wang.

**Methodology:** Songlang Liu, Xia Li, Jiewen Wei.

**Investigation:** Jiewen Wei.

**Project administration:** Yunhao Wang.

**Resources:** Yunhao Wang.

**Software:** Xia Li.

**Supervision:** Yunhao Wang, Jiewen Wei.

**Validation:** Xia Li, Jiewen Wei.

**Visualization:** Songlang Liu.

**Writing – original draft:** Songlang Liu, Xia Li, Yunhao Wang.

**Writing – review & editing:** Jiewen Wei.
